# Anthracycline Chemotherapy and Cardiotoxicity

**DOI:** 10.1007/s10557-016-6711-0

**Published:** 2017-02-09

**Authors:** John V McGowan, Robin Chung, Angshuman Maulik, Izabela Piotrowska, J Malcolm Walker, Derek M Yellon

**Affiliations:** The Hatter Cardiovascular Institute, University College London, London, WC1E 6HX UK

**Keywords:** Cancer anthracycline doxorubicin chemotherapy cardiotoxicity cardioprotection

## Abstract

Anthracycline chemotherapy maintains a prominent role in treating many forms of cancer. Cardiotoxic side effects limit their dosing and improved cancer outcomes expose the cancer survivor to increased cardiovascular morbidity and mortality. The basic mechanisms of cardiotoxicity may involve direct pathways for reactive oxygen species generation and topoisomerase 2 as well as other indirect pathways. Cardioprotective treatments are few and those that have been examined include renin angiotensin system blockade, beta blockers, or the iron chelator dexrazoxane. New treatments exploiting the ErbB or other novel pro-survival pathways, such as conditioning, are on the cardioprotection horizon. Even in the forthcoming era of targeted cancer therapies, the substantial proportion of today’s anthracycline-treated cancer patients may become tomorrow’s cardiac patient.

## Introduction

Cancer outcomes continue to improve due to earlier detection and newer targeted therapies, with anthracycline chemotherapy playing a major role in the modern era of cancer treatment. The serendipitous discovery of doxorubicin from *Streptomyces peucetius* and its precursor daunorubicin was a milestone in antibiotic overproduction techniques of the day [1]. Anthracyclines are listed among the World Health Organisation (WHO) model list of essential medicines [2]. Fifty years on from its discovery, anthracycline anti-tumour and cardiotoxic mechanisms alike continue to evoke considerable interest in basic science and clinical trials research.

Cancer now affects more than one in three people in their lifetime, and along with cardiovascular disease, they are the two leading causes of death in developed nations. Overall ten-year cancer survival stands at 50% across the twenty most common malignancies and approximately 80% or better in breast, lymphoma, melanoma and uterine cancers. These mortality trends reflect a broad improvement in survival rates in the developed economies [3]. In the United Kingdom survival trends have doubled in adults in the last 40 years and tripled in children since the 1960s [4]. Paradoxically, improved long term cancer survival has led to an increased awareness of the adverse cardiac effects of cancer treatment itself. These welcome improvements have thus shifted the care paradigm from cancer *survival* to cancer *survivorship*.

### The Role of Anthracyclines – today’s Cancer Patients Are tomorrow’s Cardiac Patients

New biological and small molecule treatments have dramatically improved the outlook of many cancers over the last 15 years [5–7]. Even in this encouraging context, anthracycline chemotherapy regimens play a prominent role in many cancer treatments – e.g.32% of breast cancer patients [8], 57 to 70% of elderly lymphoma patients [9, 10], and 50 to 60% of childhood cancer survivors are treated with an anthracycline regimen [11]. Thus, with long term *cancer survivorship* there will be a substantial population of cancer patients who will continue to remain at risk of early cardiovascular morbidity and mortality due to their legacy anthracycline chemotherapy [12, 13].

### Anthracyclines: Structure, Targets and Toxicity

**Fig. 1:**
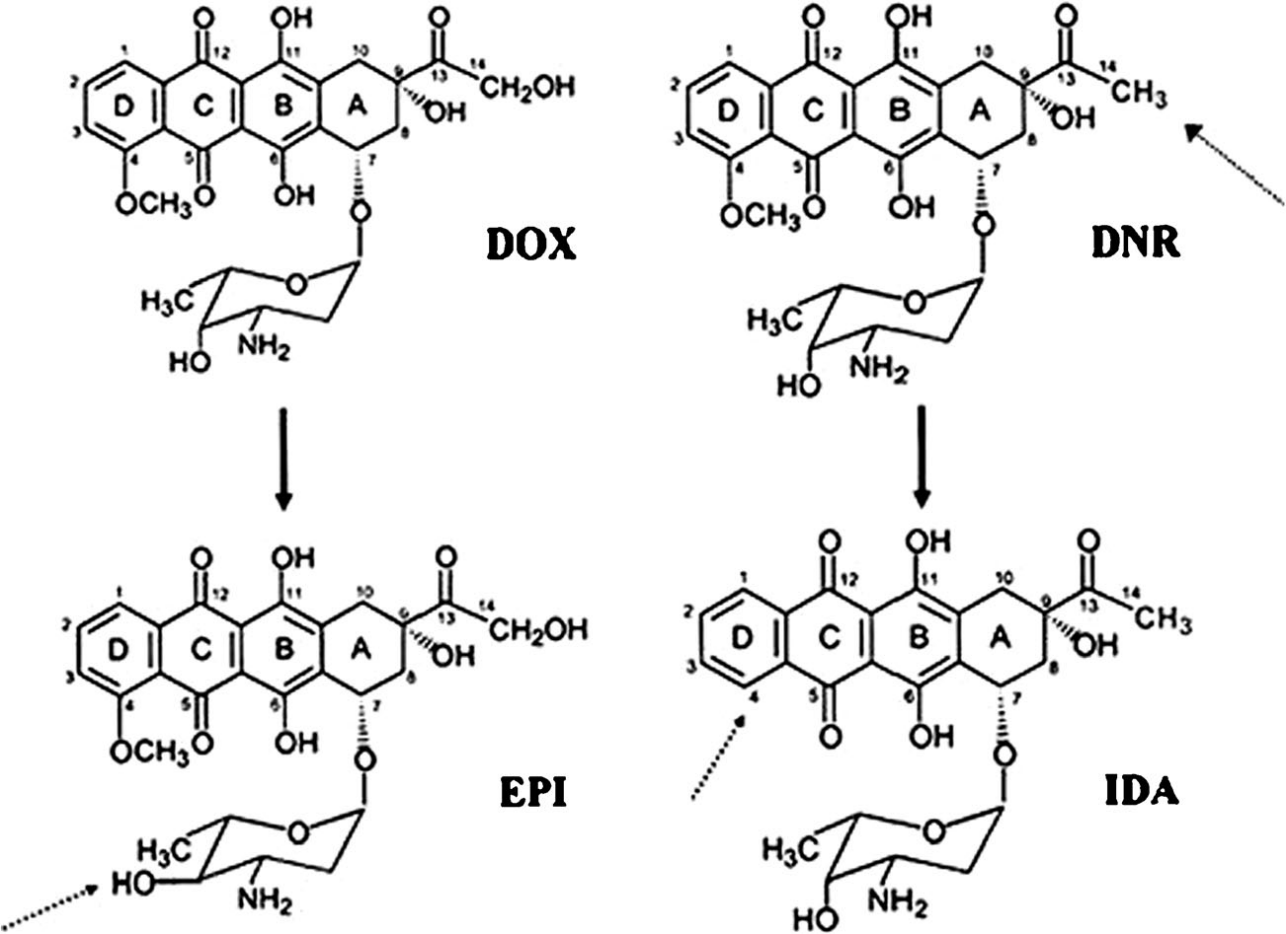
The four anthracycline derivatives Doxorubicn (DOX), Daunarubicin (DNR), Epirubicin (EPI) and Idarubicin (IDA). The anthracyclines share a tetracyclic aglycone structure of four cyclohexane chains with a daunosamine sugar moiety at carbon C7 of ring **a**; adjacent quinone-hydroquinone groups in rings **b** and **c**; a methoxy substituent carbon C4 in ring D; a carbonyl group at C13; and a short side chain in C9. Doxorubicin and daunorubicin differ in their short chains. Doxorubicin has a primary alcohol, whereas daunorubicin has a methyl group. Epirubicin is derived from doxorubicin by axial-to-equitorial epimerisation of the hydroxyl group in the daunosamine moiety. Idarubicin is identical to daunorubicin except the 4-methoxy group in ring D is removed

The four most common anthracyclines are doxorubicin, daunorubicin, epirubicin and idarubicin (Fig. 1). Doxorubicin and daunorubicin were the first to be used in clinical practice. Epirubicin, a stereoisomer of doxorubicin, has an increased volume of distribution and longer half-life than doxorubicin (doxorubicin t½ = 1–3 h, epirubicin 31–35 h). Idarubicin, a derivative of daunorubicin, is more lipophilic and has a higher cellular uptake than daunorubicin.

### Mechanisms of Action

**Fig. 2:**
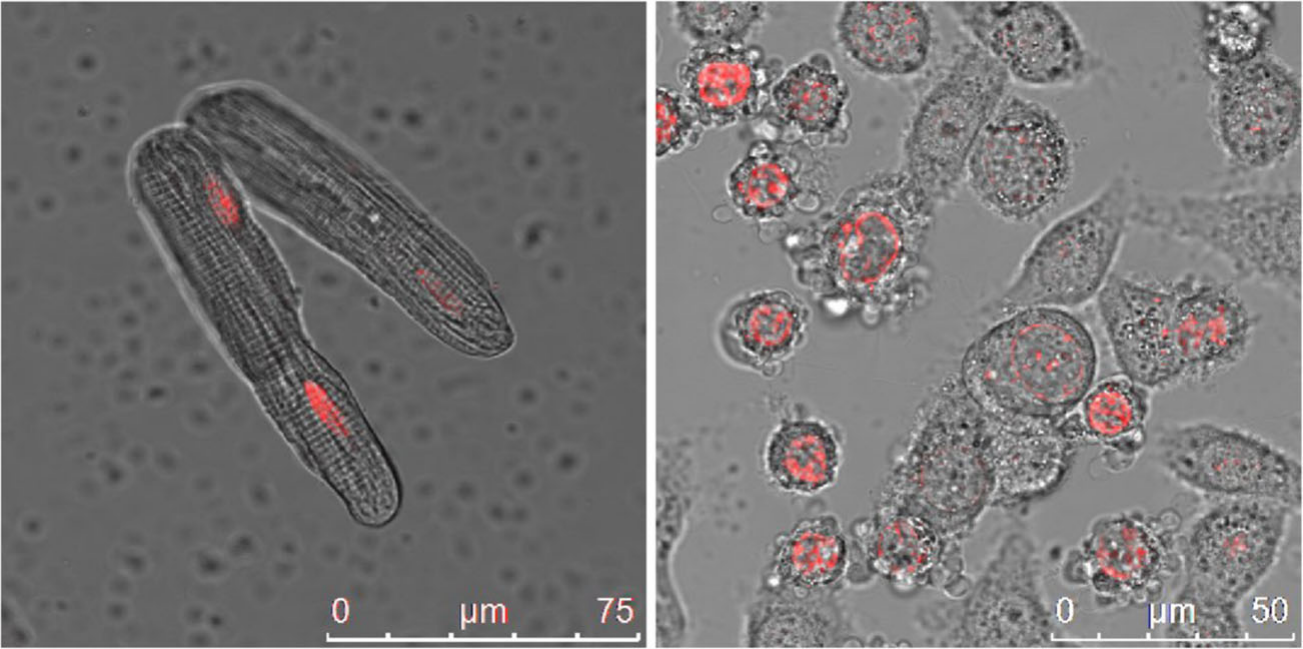
Doxorubicin staining shows sequestration in (L) cardiomyocytes and (R) malignant cervical cancer cells (courtesy Dr. I Piotrowska)

The exact mechanism of anthracycline-induced cardiotoxicity remains unclear, though it is likely to be multifactorial. Until recently, the most widely accepted hypothesis was that anthracyclines interfered with redox cycling, resulting in DNA damage due to reactive oxygen species (ROS) production [14]. More recently, topoisomerase 2 has been suggested to be the main mediator of cardiotoxicity, though other mechanisms contribute. (Fig. 2)

#### The Role of Reactive Oxidation Species

The quinone moiety of anthracyclines are susceptible to univalent reduction to a semiquinone radical by a number of cellular oxido-reductases. In myocardial cells, this is predominantly achieved via an enzymatic pathway involving NADH dehydrogenase (complex I) of the mitochondrial electron transport chain [15]. In the presence of molecular oxygen, the semiquinone auto-oxidises to generate the parent anthracycline and a superoxide anion [16]. This non-enzymatic pathway allows a self-perpetuating redox cycle to be established, resulting in the accumulation of superoxide anions. ROS levels may also be increased by free cellular iron and potentiating ferrous-ferric cycling of molecular iron [17]. The doxorubicin-iron complexes form toxic radical and reactive nitrogen species, resulting in increased nitrosative stress and mitochondrial dysfunction [18].

#### The Role of Topoisomerase 2β

DNA topoisomerases (Top) induce temporary single or double-stranded breaks to regulate the topological changes during DNA replication, transcription, recombination and chromatin remodelling [19]. In humans, Top2 is expressed as isoenzymes Top2α and Top2β [20]. Top2α is the most prevalent and is highly expressed in proliferating (malignant and non-malignant) cells. It is essential for chromosomal segregation and its expression varies during the cell cycle, peaking during G2/M phases [21]. Conversely, Top2β is more abundant in quiescent cells, such as adult mammalian cardiomyocytes, and its expression remains constant throughout the cell cycle.

Doxorubicin exerts its cytotoxic effect by intercalating DNA. Doxorubicin binds with DNA and topoisomerase 2 isoenzmes forming a ternary Top2-doxorubicin-DNA complex, which causes double-stranded DNA breaks. When bound to Top2α, the complex inhibits DNA replication; arrests the cell cycle in G1/G2; and induces apoptosis [22] as intended in proliferating malignant cells. Conversely, when bound to Top2β, mitochondrial dysfunction is triggered by the suppression of peroxisome proliferator-activated receptor (PPAR), which regulates oxidative metabolism [23], In adult mammalian cardiomyocytes, this leads to an activation of altered P53 tumour suppressor pathway, β-adrenergic signalling, impaired calcium handling, mitochondrial dysfunction and increased apoptosis. Without Top2β, doxorubicin cannot bind directly to DNA [22]. Animal studies with Top2β knockout (KO) mice have shown that the absence of Top2β protects against doxorubicin-induced cardiotoxicity [24, 25] partially due to reduced mitochondrial dysfunction [25].

#### ErbB2/ErbB4 and NRG-1 Signalling

Anthracyclines may also disrupt neuregulin-ErbB (NRG) receptor signalling to downstream cardiomyocyte pro-survival cascades involving the serine-threonine protein kinase B / AKT (whose origins stem from two human homologues of the retroviral oncogene *v-akt* [26]), mitogen-activated protein kinase (MAPK), phosphoinositide 3-kinase (PI3K) and extracellular signal-related kinase (ERK1 / 2). NRG-1 controls cell fate decisions in neuronal, skeletal myocyte, glial cells and cardiomyocytes. The ErbB complexes themselves are important modulators of cell growth. Overexpression of the NRG-1 receptor subunit ErbB2 is an important driver in certain neoplasms dependent on ErbB3 and ErbB2 (i.e. HER2+ breast cancer). NRG-1 also binds ErbB4 and stimulates proliferation of differentiated mononucleated cardiomyocytes, leading to ErbB2/ErbB4 heterodimerization [27]. This leads to compensatory cellular hypertrophy, whereas deletion models demonstrate dilated cardiomyopathy and systolic dysfunction in the presence of pressure overload [28]. Anthracyclines appear to increase ErbB2 expression after chronic doxorubicin exposure, but decrease ErbB4 expression after acute doxorubicin dosing, potentially explaining the time course of anthracycline cardiomyocyte toxicity [18].

#### Other Targets

The Ras-related C3 botulinum toxin substrate 1 – guanosine triphosphate enzyme (Rac1 GTPase) subunit also contributes to anthracycline cardiotoxicity by fragmenting histone-associated DNA and activating the programmed cell death caspase-3 (cysteine-aspartic acid protease) enzyme via nicotinamide adenine dinucleotide phosphate (NADPH) oxidase activation and ROS [29]. Other mechanisms include decreased c-Kit + cardiac progenitor cells following juvenile anthracycline exposure, leading to impaired compensatory mechanisms for stress and vascular injury repair, [30] and myofibril instability due to titin proteolysis and increased calcium sequestration, which may lead to diastolic dysfunction [28]. Together, these two mechanisms may partly explain the late cardiotoxicity seen in childhood cancer survivors manifest as early dilated cardiomyopathy followed by a period of compensated systolic function before a decompensated phenotype of restrictive physiology, decreased myocardial wall thickness and ejection fraction. [31].

## Cardiotoxicity – in Search of A Consensus Definition

The cardiovascular side effects of anthracycline anti-tumour agents became apparent soon after their widespread use in the 1970s, but the more recent concept of ‘cardiotoxicity’ has no standard definition. The mechanisms, detection, prevention, and protection from anthracycline cardiotoxicity remain relevant today because of its long legacy of treated patients and because cumulative dose-related toxicity constrains treatment. We deconstruct ‘cardiotoxicity’ according to patient age, time course, and affected cardiac domain (clinical decompensation, structural change, biomarker rise, or arrhythmia).

### Incidence

The incidence of cardiotoxicity varies according to how it is defined. Cardiotoxicity spans a continuum of frequency and severity, ranging in seriousness from overt clinical symptoms requiring urgent hospital admission, to an asymptomatic detectable structural change on cardiac imaging or new onset arrhythmia, to a measurable biomarker rise before symptomatic, structural or electrical change is detectable. These sub-types of cardiotoxicity occur as clinical decompensation in 2–4%, sub-clinical structural change in 9–11%, arrhythmia (e.g. AF) in >12%, and biomarker rise in 30–35% of cancer chemotherapy patients.

Cardiotoxicity, first observed in adult cancer patients as clinical congestive heart failure (CHF), characterised by pulmonary oedema, fluid overload, and effort intolerance, was initially reported in 1979 by Von Hoff et al. at 2.2% overall with a cumulative doxorubicin dose-dependent incidence of CHF of 3%, 7%, and 18% at 400, 550, and 700 mg/m^2^, respectively [32]. A further retrospective pooled analysis of three breast and lung cancer trials by Swain et al. documented higher rates of CHF – 4.7%, 26%, and 48% at 400, 550 and 700 mg/m^2^, respectively **-** providing the current basis to limit lifetime cumulative doxorubicin-equivalent exposure to ≤450 mg/m^2^ in patients without prior chest radiotherapy [33]. Further studies of biopsy-confirmed doxorubicin cardiomyopathy documented alarmingly poor prognosis – hazard ratio for fatal cardiotoxicity 3.46 and 50% mortality at two years – akin to restrictive and infiltrative cardiomyopathies [34].

Sub-clinical cardiotoxicity is commonly defined on cardiac imaging as clinically asymptomatic left ventricular systolic dysfunction (LVSD) with a fall in left ventricular (LV) ejection fraction (EF) by >10% points to a value of EF < 50% [35, 36]. When defined as radionuclide MUGA ejection fraction (EF) fall of more than 10% to an absolute value <50%, cardiotoxicity occurred with a cumulative incidences in 9%, 18%, 38%, and 65% of patients at 250, 350, 450 and 550 mg/m^2^, respectively, and Schwartz et al. classified 14.8% (220 of 1487) doxorubicin-treated cancer patients as high risk on the basis of abnormal falls in MUGA LVEF prior to the onset of clinical congestive cardiac failure, thereby establishing LVEF as predictive of clinical heart failure. A dose-escalation curve and reduction in %EF by MUGA scan were reported in breast cancer patients by Speyer et al., − 4% at 275–400 mg/m^2^, 15% at 400–500 mg/m^2^, 16% at 500–699 mg/m^2^, 18% at 800–899 mg/m^2^, and 21% at 900–999 mg/m^2^.

Smith et al. [11] reported significantly increased odds ratio for clinically symptomatic (OR 5.4 [95% CI 2.3–12.6]) and sub-clinical fall in LVEF (OR 6.25 [CI 2.58–15.13]) for anthracycline cardiotoxicity in their meta-analysis of 55 anthracycline cancer trials. Hequet et al. [35] documented a 27% incidence of 5-year sub-clinical cardiotoxicity in 39 of 141 adult lymphoma patients (mean doxorubicin dose 300 mg/m^2^, mean age 54 years), using echocardiographic criteria of fractional shortening (FS) < 25% (equivalent to EF < 50%). Predictors for sub-clinical cardiotoxicity were increasing age, male sex, doxorubicin dose and being overweight.

In the modern contemporary series by Cardinale et al., cardiotoxicity (defined as an echocardiographic LVEF decrease of more than 10% to EF < 50%) occurred in 9% of a mixed group of 2625 adult patients (aged 50 ± 13 years, 51% breast cancer, 28% Hodgkin lymphoma, 74% women) followed up for a median period of 5.2 years [37]. Of note, 98% of cases of asymptomatic LVEF decline were detected prospectively in the first 12 months of follow-up after chemotherapy, implicating early cardiotoxicity rather than a “late effects phenomenon” in adults as the dominant natural time course of anthracycline chemotherapy.

Relying solely on LVEF as a sub-clinical imaging marker of cardiotoxicity is problematic. The measurement itself is calculated by various echocardiographic techniques, each with its own limitations. Thavendiranathan et al. reported the minimum detectable change in EF by the same observer was 9%, 10%, and 4.8% by 2D bi-plane, tri-plane, and 3D methods; whereas the minimum detectable change in EF by different observers on different acquisitions (interobserver test-retest) was 13%, 16%, and 6% for 2D bi-plane, tri-plane and 3D methods [36]. Thus, the reproducibility and precision of echo-based LVEF measurement may be worse than the “asymptomatic 10% fall in EF” which is commonly used as the decision threshold to define cardiotoxicity (Table 1).

**Table 1:** Equivalent anthracycline dosages relative to doxorubicin (adapted from [38])

Anthracycline	Relative cardiotoxicity	Incidence of LVSD / HF
Doxorubicin	1	3–5% at 400 mg/m^2^
Epirubicin	0.7	0.9–11.4% at 900 mg/m^2^
Idarubicin	0.53	5% at 150 mg/m^2^
Liposomal doxorubicin	0.5	2% at 900 mg/m^2^

These limitations have led to development of more reliable measurements based on myocardial deformation that detect early structural change before a fall in EF. A relative change in 2D speckle-tracking echocardiographic global longitudinal strain (GLS) of 10 to 15% (95% CI = 8.3% - 14.6%) or an absolute GLS “greater” than −19% predicted cardiotoxicity defined as a fall in EF with sensitivity of 65–86% and specificity of 73–95%.

### The Role of Cardiac Troponins

Cardiac troponin is central to the universal definition of acute coronary syndrome (ACS), and its release is predictive of prognosis in myocardial infarction and other forms of heart disease [39–46]. When measured as an early biomarker of cancer cardiotoxicity, troponin rise occurs consistently in 21% - 40% of patients after anthracycline chemotherapy, irrespective of assay type [47–49]. Though troponin-I and troponin-T levels are not directly comparable across the available assays, representative levels ranged from 11 to 120 ng/L in low-dose, and 160 to more than 1900 ng/L in high-dose anthracycline regimens (see Table 2) [56]. In the setting of cancer chemotherapy cardiotoxicity, the rise in troponin may quantify both cardiomyocyte apoptosis and myofibril degradation more than necrosis and predicts cardiotoxicity defined as fall in LVEF as well as major adverse cardiovascular events (MACE).

**Table 2:** This table illustrates the wide variation in troponin-I and troponin-T assays, troponin values, and study sizes. Although not directly comparable, we have converted the levels to [ng/L] here for simplicity. Peak troponin values in high-dose chemotherapy studies reached 1980 ng/L; peak values in low-dose studies 11–120 ng/L; study sizes ranged from 19 to 703 participants. Studies routinely classified 30–40% of patients as ‘troponin-positive’ across various generations of troponin assays with differing cut off values. (TnI = troponin-I, TnT = troponin-T)

Study	Patient mix	M:F Age	Trop+/ sample size (% positive)	Baseline Trop+	Tn values (ng/L)	Troponin Cut off (as ng/L)	Troponin assay
Cardinale 2000 [50]	Advanced cancer high dose chemo^i^	39:165 45 ± 10y	65/204 (32%)	0%	1000 ± 400 Delta EF -18%	500	Stratus II TnI
Cardinale 2002 [51]	Breast CA with HDC^2^	211 F 46 ± 11y	70/211 (33% )	0%	900 ± 500	500	Stratus II TnI
Cardinale 2004 [48]	Advanced cancer with high dose chemo	216:487 47 ± 12y	208/703 (30% )	0%	E: 160 ± 240 Event 1%, 37%, 84% See below	80	Stratus CS Tn I
Sandri 2003 [52]	Advanced cancer high dose chemo	42:137 47 ± 11y	(32% ) Delta EF −18%	1%	tn + 630 ± 540 [80–1980] tn neg =39 ± 19	80	Stratus II CS Tn I
Auner 2003 [47]	Haematological adults	32:46 58y	78 (15% ) Delta EF >10%	0%	Med 40; [30–120]	30	Roche ElecIII TnT
Lipshultz [53]	ALL children Dox v Dex + Dexraz RCT	120:86 7.4 y	55/158 (35%).	12/119 ^a^ (10%)	Tn + 50% Tn++32% Dex 21%, 10%	10 + 25++	Roche ElecsysTnT
Kilickap 2005 [49]	Advanced haem CA with high dose chemo	20:21 44y	(34%)	*N* = 1 (16 ng/L)		10? 100 Error in paper	Roche ElecIIITnT
Haney [54]	Breast CA	22 F	41% (9/22)	n/a	Peak 60 ng/L Cycle 6: 50% Tn+	Tn+ > 12 ng/L; TnT+ 22/ samples 91	Roche TnT
Katsurada [55]	Breast CA anthracycline + Herceptin	19F only Age N/A	*N* = 19 hsTnT values	N/A	11 +/− 7.8 4 +/− 1.4	14	Roche hs-TnT

Baseline troponin levels may be elevated due to the burden of malignant disease itself. Auner et al. reported a baseline troponin-T levels >70 ng/L in 3.8% of 78 mixed acute myeloid and non-Hodgkin lymphoma patients [57]. Lipshultz et al. documented baseline increased troponin-T levels >10 ng/L in 10% of 119 paediatric acute lymphoblastic leukaemia, associated with a ten-fold increase in white-cell count (300 × 10^3^/mm^3^ vs. 27 × 10^3^/mm^3^) and significant blast crisis (89% vs. 57.5%) [53], reflecting the cardiotoxic effects of profound leucocytosis in the circulation, and in particular, the coronary circulation even before anthracycline exposure [58–60]. Cardiac troponin-T levels in the unprotected control cohort of the study were elevated in 50% of 76 patients, with multiple troponin-T elevations occurring in 37%.

Cancer itself may be arrhythmogenic, and its treatments may increase supraventricular tachyarrhythmias and atrial fibrillation [61, 62]. Baseline AF incidence in cancer was reported as 2.4% and new onset AF occurred in a further 1.8% in a large cohort of 24,125 cancer patients [63]. Early short term arrhythmia studies documented increased ventricular ectopy at 1 h post-infusion in 3%, rising to 24% during 1 to 24 hours of Holter monitoring.

### Time Course of Cardiotoxicity

The time course of cardiotoxicity varies depending on patient age at time of exposure and the class effect of chemotherapy drugs, where childhood cancer survivors experience exponentially rising risk for cardiovascular events (a “late effect”), but older adults’ cardiovascular risk manifests earlier and is dependent on the number of conventional co-existing cardiac risk factors — hypertension in particular.

Kero et al. [64] reported increased hazard ratios (HR) 3.6 and 13.5 times that of age-matched sibling controls for cardiomyopathy in cancer survivors aged 0–19 years and 20–34 years, respectively, as well as HR 3.3 and 1.8 for ischaemic heart disease in the same age groups, respectively. Armstrong et al. have defined a long period of latency in childhood and young adult cancer survivors of 20 or more years in longitudinal cohort studies. Further evidence of individual cardiac risk and premature cardiac ‘ageing’ was documented in breast cancer survivors (treated with a mix of anthracyclines and radiotherapy). Peak oxygen consumption scores in breast cancer survivors were equivalent to scores in controls *20 years older* – mean VO2 peak (mVO2) = 19.5 ml/kg/min in breast cancer survivors aged 50 years compared to mVO2 = 19.3 ml/kg/min in a cohort of healthy 70 year old controls [65]. Thus evidence of early cardiac aging in individual cancer survivors bears striking similarities to the epidemiological outcomes in adult survivors of childhood cancers.

### Childhood Cancer Cardiotoxicity

Long-term outcomes in children treated for acute lymphoblastic leukaemia with or without dexrazoxane cardioprotection document a time course of sub-clinical left ventricular disease that is distinct to that of adults [66]. Cancer-treatment related cardiotoxicity is increased in younger (especially age < 7 years), female patients, chest radiotherapy, higher doses of anthracyclines, as well as higher body fat composition. The reasons for this are not completely understood. Anthracycline-mediated reduction in C-kit + cardiac progenitor cells resulting in impaired myocardial growth (and reduced LV mass) during increased somatic growth may amplify cardiotoxic vulnerability from the loss of myocytes during anthracycline exposure in childhood [67]. Autopsy studies in human hearts have documented preferential accumulation of doxorubicin in cardiac over skeletal and smooth muscle with gradual conversion to doxorubicinol [68]. Paediatric cancer treatments may be particularly cardiotoxic as neonatal murine heart mitochondria appear ‘primed’ for apoptosis via the B-cell lymphoma 2 / BCL-2 homology (BCL2-BH3) pathway compared to apoptosis-refractory adult mitochondria [69]. These putative mechanisms, together with diastolic dysfunction from increased calcium sequestration and titin proteolysis, may partially explain why paediatric cancer survivors demonstrate a decompensated phenotype after anthracycline exposure. Childhood cancer survivors demonstrate progressive sub-clinical left ventricular disease, as somatic growth outpaces paediatric cardiomyocyte growth, manifest as early dilated cardiomyopathy followed by a period of compensated systolic function and later restrictive physiology (normal to low cavity size, reduced ventricular wall thickness and fractional shortening), a distinctly different time course compared to adult cancer survivors.

### Controversies in Cardiotoxicity: Type 1 Vs. Type 2 and the Early Vs. Late Paradox

The concept of reversible (‘type 1’) and irreversible (‘type 2’) cardiotoxicity was proposed with the introduction of HER2/ ErbB receptor blockers such as trastuzumab (Herceptin ®) [70]. Here the argument was that anthracycline cardiotoxicity was persistent, dose-related and irreversible (type 1), whereas trastuzumab cardiotoxicity was reversible, not dose-related and LV ‘function’ recoverable upon discontinuation of trastuzumab. This concept has not been without controversy because some cardioprotection studies suggest that anthracycline cardiotoxicity maybe reversible if detected within 3–6 months of a decline in ejection fraction [71, 72]. Furthermore, the typical regimen of trastuzumab was administered concomitantly with anthracyclines, though currently it is administered sequentially after anthracyclines, thereby confounding any lone effect of trastuzumab with anthracycline exposure.

Cardinale et al. prospectively followed 2625 cancer patients and documented a single linear time course for anthracycline cardiotoxicity defined as a fall in LVEF [37]. In this cohort, followed up for a median of 5.2 years, 98% of cardiotoxicity was documented in the first 12 months’ follow-up, without a bimodal “early” and subsequent “late effects” distribution, contrary to conventional wisdom – casting dispersions on the widely held belief of ‘early’ and ‘late’ cardiotoxicity in adult cancer survivors. Cardinale argued against the classic ‘late’ cardiotoxicity paradigm in adults, because prior studies detecting late phenomena were retrospective, and instead proposed that ‘late’ cardiotoxicity was ‘early’ cardiotoxicity that had escaped detection at an earlier stage.

## Outcomes

The long term cardiovascular outcomes of cancer survivors vary according to several risk profiles. The predictors for cardiovascular outcomes after cancer survival include age at time of diagnosis, female gender in childhood cancer survivors, chest radiotherapy, time since cancer treatment [13] and anthracycline dosage [73]. Kero et al. reported increased HR for cardiomyopathy =4.6 (and strikingly high HR 13.5 for age 0–19 y) and ischaemic heart disease =1.8 compared to sibling controls for young adult cancer survivors aged <35 years.

The importance of cardiology follow-up for late cardiotoxicity due to chemotherapy is particularly important in childhood cancer survivors. The cumulative incidence of adverse cardiovascular outcomes by age 45 years in 10,724 childhood cancer survivors (acute lypmphoblastic leukaemia [ALL] 30%, Hodgkin Lymphoma [HL] 12.8%, Wilms tumour 9.6%, sarcoma 8.7%) are strikingly higher [73]. The incidence in childhood cancer survivors compared to sibling controls of coronary artery disease, heart failure, and valvular disease was 5.3% vs. 0.9%, 4.8% vs 0.3%, and 1.5% vs. 0.1%, respectively, signifying an increased relative risk (RR) between 6 and 16. Hypertension alone was associated with an increased RR of 5.6 of cardiac death. This substantial body of long term follow-up has produced an individualized childhood cancer survivor risk calculator [74].

Cardiotoxic morbidity and mortality occur at higher numerical epirubicin doses than for doxorubicin. Dose-related NYHA class II or worse congestive heart failure (CHF) incidence occurred in 1.9% at 800 mg/m^2^, 4% at 900 mg/m^2^, and 15% at >1000 mg/m^2^ in a series of 469 epirubicin-treated breast cancer patients [75]. CHF occurring at a median onset of 57 days with overall 38% mortality and median survival of 162 days and 125 days if patients had received radiotherapy.

Cardiac mortality is an increasingly important determinant of overall prognosis after cancer. Cardiovascular death was the leading cause of death surpassing recurrent breast cancer (15.9% vs. 15.1%) after median nine-year follow up in 63,566 female breast cancer patients [13]. Women were more likely to die of cardiovascular causes than from breast cancer as they aged, with cardiac death overtaking cancer death after nine years survivorship. In childhood cancer survivors, cardiac deaths accounted for 13% of deaths after 45 years’ survival, and the annualised cardiac death rate exceeded that of cancer recurrence (0.14% vs. 0.05%) after 30 years’ survivorship [76]. Thus with ever longer cancer survivorship, cardiovascular mortality supervenes cancer mortality.

### Pregnancy Outcomes

Cancer is the second most common cause of death during the female reproductive years and it occurs in 1 in 1000 pregnancies with breast, cervical, lymphoma and leukaemia’s the most common malignancies [77]. Lymphoma occurs in 1 in 6000 pregnancies, and identified risks include increasing maternal age and HIV-related non-Hodgkin’s lymphoma in developed and developing countries, respectively [78]. Termination of pregnancy is recommended when malignancy is diagnosed during the first trimester due to the likelihood of aneuploidy and disrupted organogenesis, but until recently malignancy management in later pregnancy was based on small case report data. Pinnix et al. reported promising 5-year long term outcomes in lymphoma (HL and NHL) diagnosed during pregnancy. In a retrospective series of 39 pregnant women (median age 28 years), there were three terminations. In the remaining 36 pregnancies, 24 elected to have antenatal chemotherapy and 12 elected to defer treatment until after delivery. There were 4 miscarriages (two in first trimester, all four in the antenatal chemotherapy group), and the median gestational age of delivery was 37 weeks. There were no reported foetal anomalies. Progression free survival at 5 years was 74.7%, (overall survival =82.4%). This compared favourably with 5-year HL survival of 86% and NHL of 69.5% in women who were not pregnant [4].

### The Search for Cardioprotective Therapies

A number of potential cardioprotective techniques and therapies have been explored ranging from modified anthracycline preparations, anti-oxidants, free radical scavengers, renin-angiotensin-system antagonists, cardioselective beta-blockers to statins. Whilst many show promise in animal studies, the clinical studies have had mixed results.

Modified analogues of doxorubicin such as epirubicin and liposomal doxorubicin are relatively less cardiotoxic than conventional doxorubicin. Epirubicin was associated with decreased odds ratio for clinical 0.39 (95%CI 0.20–0.78) and sub-clinical fall in LVEF (OR 0.30 (95% CI 0.16–0.57) [11]. In a meta-analysis, epirubicin had lower rates of clinical cardiotoxicity (OR 0.39, 0.2 to 0.78; *p* = 0.008; I^2^ = 0.5%) and subclinical cardiotoxicity (OR 0.30, 0.16 to 0.57; *p* < 0.001; I^2^ = 1.7%) than doxorubicin without compromising anti-tumour efficacy [11]. Liposomal doxorubicin also had lower rates of clinical cardiotoxicity (OR 0.18, 0.08 to 0.38; *p* < 0.001; I^2^ = 0%) and subclinical cardiotoxicity (OR 0.31, 0.20 to 0.48; *p* < 0.01; I^2^ = 48.5%) than doxorubicin, again, without compromising efficacy. Liposomal doxorubicin and epirubicin have similar levels of cardiotoxicity (RR 1.15, 0.47 to 2.84; *p* = 0.754) [11]. Continuous infusion doxorubicin was compared against bolus infusion in children with acute lymphoblastic leukaemia. There were similar levels of LV systolic dysfunction, cavity dilatation, reduced wall thickness and LV mass at 8 years, and thus no cardioprotection in children [79].

N-acetylcysteine has antioxidant properties and was therefore hypothesised to be of benefit due to the ROS hypothesis. However, clinical studies have failed to demonstrate a benefit from N-acetylcysteine in preventing or reversing doxorubicin-induced cardiomyopathy. In a study of doxorubicin-naïve patients with normal left ventricular function, patients given N-acetylcysteine 1 h before their first dose of doxorubicin had similar histological damage (tubular and mitochondrial area) on endomycardial biopsies taken at 4 and 24 h after doxorubicin administration to controls. In a small randomised controlled trial of 19 disease-free sarcoma patients with doxorubicin-induced cardiomyopathy, patients receiving N-acetylcysteine 5.5 g/m^2^ daily for 30 days (*n* = 11) showed no difference in left ventricular ejection fraction at rest or during exercise compared to controls [80]. The largest randomised controlled trial was performed by Myers et al. who randomised 54 patients with breast cancer, nodular lymphoma or metastatic soft tissue sarcoma to receive either N-acetylcysteine 5.5 g/m^2^ orally prior to doxorubicin or placebo. The rates of clinical heart failure were similar between the intervention and control groups (12.5% vs 10%, *p* = 0.77) [81].

Calcium antagonists have been studied in two small clinical trials. Milei et al. studied prenylamine in 26 adults with solid tumours undergoing doxorubicin chemotherapy. One patient in the control group (*n* = 13) and no patients in the intervention group (*n* = 13) developed cardiomyopathy [82]. Kraft et al. studied low-dose verapamil in adult patients with acute myeloid leukaemia [83]. One patient in the control group (*n* = 17) and no patients in the intervention group (*n* = 13) developed heart failure (no significant difference).

Amifostine, a broad-spectrum cytoprotective agent, showed promise in animal models, where it prevented doxorubicin-induced cardiotoxicity [84, 85]. However, this did not translate in a clinical study of 28 children with osteosarcoma treated with cisplatin and doxorubicin, where no cardiac benefit was observed. Moreover, amifostine was poorly tolerated, with 93% of patients suffering grade 3/4 vomiting compared to 7% in the control group.

Coenzyme Q10 was studied in a mixed cohort of 20 children with acute lymphoblastic lymphoma (ALL) and HL. Children in the control group had a reduction in interventricular septum wall thickening, measured by echocardiography, following anthracycline chemotherapy. The authors suggested a protective effect from coenzyme Q10, though this has not been replicated in larger studies [86]. Despite their promise for providing cardioprotection, free radical scavengers have failed to show protection in clinical studies. Probucol, a lipid-lowering agent and antioxidant, has shown to be protective against doxorubicin-induced cardiomyopathy without compromising antitumour efficacy in rats and mice.

Animal studies using dexrazoxane had mixed results in vivo. Deferiprone (Ferriprox) demonstrated anthracycline cardioprotection in a rat model [87, 88]. However, dexrazoxane is the only iron chelator that has been licenced for clinical use in (breast) cancer patients undergoing extended anthracycline dosing in excess of 300 mg/m^2^.

Dexrazoxane’s cardioprotective mechanism against anthracyclines was considered to be due to iron chelation, preventing anthracycline-iron binding and the subsequent ROS formation. However, cardioprotection seems to be exclusive to dexrazoxane and deferriprone rather than a class effect as other iron chelators, such as deferasirox, have not shown cardioprotection [89]. Dexrazoxane binds to topoisomerase-2, with cardioprotection conferred via top-2β, but the role of top-2α also formed the controversial hypothesis for potentially reduced anti-tumour efficacy and its role in malignant proliferation.

Dexrazoxane has largely been evaluated in women with advanced breast cancer and adults with sarcoma, and its current approval is for extended anthracycline dosing. A meta-analysis showed that dexrazoxane administration alongside doxorubicin or epirubicin reduced the rates of clinical cardiotoxicity (OR 0.21, 0.13 to 0.33; *p* < 0.000 l; I^2^ = 0%) and subclinical cardiotoxicity (RR 0.33, 0.20 to 0.55; *p* < 0.0001 l I^2^ = 0%) compared to doxorubicin or epirubicin alone [11]. Asselin et al. reported the cardioprotective effects of dexrazoxane vs doxorubicin-only in 537 children and adolescents with T-cell acute lymphoblastic leukaemia (T-ALL) or advanced lymphoblastic Non-Hodgkin lymphoma (NHL) [90]. At three years, LV function assessed by fraction shortening was significantly lower in the doxorubicin-only group than the dexrazoxane group (*p* = 0.05). Five-year event free survival and rate of secondary malignancies was not statistically different between the dexrazoxane group and doxorubicin-only group (76.7 ± 2.7% vs 76.0 ± 2.7%, *p* = 0.9, and 0.8 ± 0.5% vs 0.7 ± 0.5%, *p* = 0.17, respectively). Further meta-analysis of five randomised control trials suggested dexrazoxane was associated with a borderline increase in secondary malignant neoplasms that was not statistically significant (absolute incidence 2.7% [17/635] vs. 1.1% [7/610], *p* = 0.06).

In addition to their lipid-lowering effects, statins also have anti-inflammatory and anti-oxidant effects [91]. They have been postulated to increase anti-tumour efficacy but clinical studies in malignant melanoma and colorectal cancer have not observed this. In a small trial of patients undergoing anthracycline-containing chemotherapy regimes, patient were randomised to receive atorvastatin 40 mg (*n* = 20), commenced before their chemotherapy and continued for 6 months, or control (*n* = 20). The primary endpoint was reduction in LVEF to <50%. One patient (5%) in the statin arm and five patients (25%) in the control arm developed LVEF <50%, however, this did not reach statistical significance (*p* = 0.18). The mean change in LVEF, left ventricular end-diastolic diameter and end-systolic diameter (LVEDD and LVSD) from baseline to 6 months for the statin group and control group were 1.3 ± 3.8 vs −7.9 ± 8.0%, *p* < 0.001; −0.15 ± 4.0 vs 2.0 ± 3.3 mm, *p* = 0.021; and −1.35 ± 4.0 vs 2.1 ± 1.8 mm, *p* < 0.001, respectively [92]. In a retrospective, observational cohort study of 201 women receiving anthracyclines for newly diagnosed breast cancer, uninterrupted statin therapy had a reduced risk of heart failure compared to propensity-matched controls (hazard ratio 0.3, 95% CI: 0.1–0.9, *p* = 0.03). However, this finding may have been confounded by a greater ACE inhibitor and β-blocker use in the statin group compared to controls (38.8% vs 17.2%, *p* < 0.001 and 38.8% vs 14.9%, *p* < 0.001, respectively) [93].

Carvedilol is a non-selective β-antagonist with some α-antagonist effect. It also inhibits NADH dehydrogenase, found in complex I of the mitochondria, and has antioxidant activity. It has therefore received considerable interest in cardioprotection against anthracycline-induced cardiotoxicity and been studied in isolation and in combination with ACE inhibitors.

The OVERCOME trial (preventiOn of the left Ventricular dysfunction with Enalapril and caRvedilol in patients submitted to intensive chemOtherapy for the treatment of Malignant hEmopathies) is the largest randomised controlled trial of combined ACE inhibitors and carvedilol [94]. Ninety adult patients with normal left ventricular ejection fraction (LVEF) and newly diagnosed haematological malignancy were randomised to either enalapril + carvedilol or control. The primary endpoints were changes in LVEF assessed by transthoracic echocardiography and cardiac magnetic resonance (CMR) at six months from baseline. The population comprised of 36 patients with acute leukaemia (acute myeloid leukaemia 30 and acute lymphoblastic leukaemia 6) and 54 were undergoing autologous stem cell transplant for Hodgkin disease (*n* = 9), non-Hodgkin lymphoma (*n* = 23), and multiple myeloma (*n* = 22). Doses of enalapril and carvedilol were similar to heart failure studies (8.2 ± 5.9 mg and 26.1 ± 18.2 mg, respectively). Left ventricular ejection fraction declined at six month in the control group on both echocardiography and CMR (−3.28% (95%CI: -5.49 to −1.07) and −3.04% (95%CI: -6.01 to −0.070, respectively), but was preserved in the intervention group (−0.17 (−2.24 to 1.90) on echocardiography and 0.36 (−2.41 to 3.13) on CMR). Interestingly, the pre-specified subgroup analysis showed that patients with acute leukaemia had a greater degree of LVEF decline, with mean − 6.4% (95%CI: -11.88 to −0.87) compared to −1.01% (95%CI: -4.46 to 2.45) in patients undergoing autologous stem cell transplant.

Prompt initiation of ACE inhibitors and β-blockers when LVEF declines is important for recovery of function [71, 95]. Cardinale et al. randomised patients with raised troponin I after high-dose anthracycline-containing chemotherapy to either enalapril (*n* = 56) or control (*n* = 58). Treatment was started one month after the completion of chemotherapy and continued for a year. The primary endpoint was a reduction in LVEF >10% to LVEF <50%. The maximum tolerated dose of enalapril was 16 ± 6 mg daily. Twenty five control subjects (43%) and no intervention subject had a reduction in LVEF to <50% (*p* < 0.001) [72]. Furthermore, their work documented that prompt initiation of treatment was an important determinant of LV functional recovery.

In their larger prospective cohort study of 2625 patients undergoin anthracycline chemotherapy, Cardinale et al. measured LVEF by serial echocardiography for a median follow-up of 5.2 years (quartile 1 to quartile 3, 2.6–8.0 years) [37]. Cardiotoxicity, defined as a reduction in LVEF of ≥10% to <50%, was seen in 226 (9%). The vast majority (98%) occurred within the first 12 months, with median time to development of 3.5 months (quartile 1 to quartile 3, 3–6 months) after completing chemotherapy. Prompt initiation of enalapril and carvedilol (*n* = 186) or enalapril alone (for those recruited before 1999, *n* = 40) at the time of detection of cardiotoxicity resulted in full recovery of LVEF, defined as return to baseline, in 25 (11%) and partial recovery, defined as an increase in LVEF ≥ 5%, in 160 (71%) patients [95]. This study also raised the intriguing observation that 98% of LV functional decline was detected in the first 12 months – challenging the paradigm of early vs. late cardiotoxicity observed in legacy retrospective studies.

Studies of carvedilol monotherapy are smaller. In adult females with non-metastatic breast cancer randomised to carvedilol (*n* = 30) or placebo (*n* = 40), commencing one week before starting doxorubicin and finishing one week after the final cycle, those taking carvedilol showed no change in their LV strain measurements from baseline to after chemotherapy, whereas patients taking placebo showed significant deterioration. Neither group showed significant deterioration in their LVEF [96]. Another small study randomised patients to carvedilol (*n* = 25) or placebo (*n* = 25) and demonstrated that the carvedilol group maintained LVEF, whereas the control group had a reduction in LVEF at 6 months compared to baseline (70.5 vs 69.37, *p* = 0.3, and 68.9 vs 52.3, *p* < 0.001, respectively).

Carvedilol has also shown a benefit in children aged 6–12 years with acute lymphoblastic leukaemia treated with doxorubicin [97]. Patients were randomised to carvedilol for five days before doxorubicin (*n* = 25) or doxorubicin alone (*n* = 25). Patients in the intervention arm had a higher fractional shortening (FS) one week after the last dose of doxorubicin compared to pre-chemotherapy (baseline 34.0 ± 4.5 vs 39.5 ± 6.3, *p* ≤ 0.05), whereas patients without carvedilol had a reduction in FS (40.0 ± 4.6 vs 33.5 ± 6.2, *p* ≤ 0.05). Diastolic parameters (E, A and E/A) were not different between the two groups. Similar findings were seen by Ewer between pre-doxorubicin and one-week after completion of chemotherapy in paediatric patients, aged 7 months to 15.5 years [98]. Nebivolol, a selective β1-antagonist with nitric oxide vasodilator properties, demonstratred increased contractility in animal studies, and cardioprotrection via preserved LVEF compared to placebo in patients with breast cancer receiving doxorubicin [99].

The recent PRADA trial (Prevention of cardiac dysfunction during adjuvant breast cancer therapy) compared the cardioproctive properties of candesartan and metoprolol in 120 patients with early breast cancer undergoing adjuvant FEC chemotherapy (fluorouracil, epirubin and cyclophosphamide) in a 2 × 2 factorial, randomised, placebo-controlled, double-blind study [100]. Patients were randomised to receive candesartan-metoprolol, candesartan-placebo, metoprolol-placebo or placebo-placebo. The primary endpoint was a change in cardiac magnetic resonance (CMR)-LVEF from baseline to the end of chemotherapy. Patients receving candesartan had no change in their LVEF (0.8, 95% CI: -0.04 to 1.9) after chemotherapy, whereas the non-candesartan group had LVEF decline of 2.6% (95% CI: 1.5 to 3.8, *p* = 0.026). No difference was seen between patients receiving metoprolol and metoprolol-naïve patients, 1.6% (95% CI: 0.4 to 2.8) and 1.8% (95% CI: 0.7 to 3.0), respectively. The PRADA results support cardioprotective properties on a statistically measurable scale, if not wholly clinically significant.

Remote ischaemic conditioning (RIC) is another potentially cardioprotective treatment that is currently under investigation in cancer patients [56]. This non-invasive non-pharmacological treatment is delivered via a blood pressure cuff as short bursts of ischaemia and reperfusion in a peripheral limb. RIC has been shown to protect the heart and other organs from subsequent severe ischaemic injury [101–107], and demonstrated lung protection in cancer surgery [103]. While the mechanism of RIC is not fully understood, it appears to involve a humoral and neural pathway that confers cardioprotection by activating innate pro-survival pathways that ultimately modulate common mechanisms in ischaemia reperfusion injury and anthracycline cardiotoxicity such as calcium overload, lipid peroxidation, ROS generation and mitochondrial function [108].

## Conclusion

The cardiotoxic mechanisms of anthracyclines may involve the dual pathways of reactive oxidation species and topoisomerase 2-beta and a final common pathway of calcium overload, lipid peroxidation and mitochondrial dysfunction. There is no universally accepted definition of anthracycline cardiotoxicity, and thus its incidence varies according to how it is defined. Clinically symptomatic heart failure occurs in 2–4%, asymptomatic fall in LVEF in 9–11%, arrhythmia in 12% or more, and cardiac biomarker rise in 30–35% of treated patients. Predictors of cardiotoxicity include cumulative dose, cardiovascular risk factors and age of treatment.

It is essential that we continue to investigate ways of protecting the heart following cancer chemotherapy, At present the limited cardioprotective strategies available -- dexrazoxane, ACE-inhibitors, ARB, and beta-blockers -- are not in routine prophylactic use. New strategies are required to help combat the cardiotoxicity derived from anthracycline and other chemotherapies with novel agents such as NRG-1 that target ErbB receptor or non-pharmacological and non-invasive techniques, such as remote conditioning, which activate endogenous pro-survival pathways in the heart.

In the forthcoming era of personalised cancer medicine and targeted therapies, it may be tempting to consign the cardiac effects of anthracyclines to history. However the large population-at-risk, long latency in childhood cancer survivors, and prospectively increased cardiac risk in adult cancer survivors have implications for cancer survivorship as a cardio-oncology partnership. Thus, the increasing cancer survivor population represents a strong motivator to explore larger randomised controlled trials in cancer cardioprotection to prevent today’s cancer patient from becoming tomorrow’s cardiac patient.
